# A Glimpse of Nucleo-Cytoplasmic Large DNA Virus Biodiversity through the Eukaryotic Genomics Window

**DOI:** 10.3390/v9010017

**Published:** 2017-01-20

**Authors:** Lucie Gallot-Lavallée, Guillaume Blanc

**Affiliations:** 1 Structural and Genomic Information Laboratory (IGS), Aix-Marseille Université, CNRS UMR7256 (IMM FR3479), 13288 Marseille cedex 09, France; lucie.gallot-lavallee@igs.cnrs-mrs.fr; 2 Mediterranean Institute of Oceanography (MIO), Aix Marseille Université, Université de Toulon, CNRS/INSU, IRD, UM 110, 13288 Marseille cedex 09, France

**Keywords:** nucleo-cytoplasmic large DNA virus, lateral gene transfer, virus insertion

## Abstract

The nucleocytoplasmic large DNA viruses (NCLDV) are a group of extremely complex double-stranded DNA viruses, which are major parasites of a variety of eukaryotes. Recent studies showed that certain eukaryotes contain fragments of NCLDV DNA integrated in their genome, when surprisingly many of these organisms were not previously shown to be infected by NCLDVs. We performed an update survey of NCLDV genes hidden in eukaryotic sequences to measure the incidence of this phenomenon in common public sequence databases. A total of 66 eukaryotic genomic or transcriptomic datasets—many of which are from algae and aquatic protists—contained at least one of the five most consistently conserved NCLDV core genes. Phylogenetic study of the eukaryotic NCLDV-like sequences identified putative new members of already recognized viral families, as well as members of as yet unknown viral clades. Genomic evidence suggested that most of these sequences resulted from viral DNA integrations rather than contaminating viruses. Furthermore, the nature of the inserted viral genes helped predicting original functional capacities of the donor viruses. These insights confirm that genomic insertions of NCLDV DNA are common in eukaryotes and can be exploited to delineate the contours of NCLDV biodiversity.

## 1. Introduction

Viruses have long been viewed only under the angle of human, animal, and plant diseases, which considerably restrained our vision of the viral world and its role in global ecology. In this age of virus discovery, we are beginning to appreciate the enormous diversity of viruses, far beyond what we originally thought. Nucleo-cytoplasmic large DNA viruses (NCLDVs) [1,2] form a monophyletic clade of eukaryotic viruses with a large double-stranded DNA (dsDNA) genome ranging from 100 kbp in the smallest iridoviruses up to 2.50 Mbp in the gigantic pandoraviruses [3]. Their hosts show a remarkably wide taxonomic spectrum from microscopic unicellular eukaryotes to larger animals, including humans [2]. The biodiversity of NCLDVs is thought to be immense however we still do not know how many major clades do exist [4]. Seven taxonomic families have been defined so far including *Ascoviridae*, *Asfarviridae, Iridoviridae, Marseilleviridae, Mimiviridae, Phycodnaviridae,* and *Poxviridae*, but new viral isolates, such as pandoravirus, pithovirus, and mollivirus, are likely to become founding members of new families. Historically, isolation of large DNA viruses infecting eukaryotic algae or protists has proceeded by co-culturing a host together with a virus sampled from the environment. In this experimental approach, a eukaryotic host is chosen a priori for its capacity of being infected by a virus and adapted to lab culture prior to virus isolation. Recently, the metagenomic approach has accelerated the rate at which new viruses are brought to light [5,6]. However, this approach suffers from two main shortcomings: first, viral sequences assembled from metagenomic data are generally short, encompassing often only a few genes at best. Second, the hosts of the identified viruses remain unknown. Yet, host information is an absolutely essential component in the study of viruses, since viral replication is dependent on host organisms [7]. Thus, drawing the contours of virus/host diversities calls for a development of new approaches that can circumvent limitations of the co-culturing and metagenomics methods.

Recent studies have identified NCLDV-related sequences in genomic and transcriptomic datasets generated from eukaryotic organisms [8,9,10,11,12,13]. Some of these viral sequences were shown to originate from virus genome fragments integrated into the nuclear genome of their presumed eukaryotic hosts, including protists [10,13], land plant [9], and algae [8,11,12,14]. These fragments encompass up to several hundreds of kbp and can contain hundreds of viral genes, including common NCLDV phylogenetic markers. It is currently unclear how and by which mechanisms these viral DNAs became integrated into eukaryotic genomes. DNA integration may result from an active process (i.e., as a result of a virus-encoded integrase activity) or from an accidental incorporation of viral DNA freely floating inside the cell (i.e., as a result of an aborted infection). Phaeoviruses are the only members of NCLDVs to show evidence of a lysogenic cycle. Presumably, they integrate into the genome of their host by means of an integrase encoded by the virus [14,15]. In addition to reports of NCLDV DNA inserts in eukaryotic hosts, NCLDV-like sequences were also found in some algal transcriptomes [11]. These transcripts may originate from viral genes integrated in the host genome or from infected host cells present in the culture from which RNA were extracted. Altogether, these studies suggested that viral DNA insertion in the host genome is a common feature of NCLDVs. However, the frequency at which this phenomenon occurs across eukaryotic lineages, and the short- and long-term evolutions of inserted viral sequences are still poorly understood. Whether they have a potential role in defense mechanisms against infecting viruses based on sequence recognition and/or RNA silencing is also an open question.

Interesting information has come out from the discovery of viral inserts: many of the organisms in which NCLDV sequences were identified were not previously known to be infected by NCLDVs. Moreover, phylogenetic markers harbored by viral genomic inserts or transcripts suggested that certain virus donors were distantly related to known NCLDVs [8,9,16]. Thus, assuming that the NCLDV sequences identified in eukaryotic datasets result from infecting viruses or lateral gene transfers, these sequences may be used as a tool to better describe the realm of NCLDVs. Importantly, identification of NCLDV genes may allow predicting novel virus/host associations and shedding new light on the biodiversity of NCLDVs. With the vertiginous throughput and dropping cost of DNA sequencing, new eukaryotic genomes are nowadays sequenced at an unprecedented pace. Thus, since the pioneering studies performed over the last couple of years [8,9,10,11,12,13,16], many new eukaryotic genomes have been released in public databases. This prompted us to perform an update survey of NCLDV genes hidden in eukaryotic sequences to measure the incidence of this phenomenon in common public databases. Here, we show that sequences generated from 66 eukaryotes contained NCLDV core genes, most of which have never been reported so far. Phylogenetic reconstruction showed that many of these sequences originated from members of existing NCLDV families, but also possibly from as yet unknown NCLDV clades, thus extending the range of the NCLDV biodiversity.

## 2. Materials and Methods

Sequences from the five NCLDV core proteins were retrieved from the NCLDV clusters of orthologous gene (NCVOG) database [17,18] and aligned against protein databases using BLASTP (E-value < 1 × 10^−5^). BLAST searches against RefSeq and 1KP databases were performed on the dedicated website at NCBI and [19]. Unannotated genome assemblies were downloaded from the NCBI Assembly database. We only downloaded the eukaryotic fraction of the assembly database to the exclusion of very large genomes (i.e., >1 Gbp) to limit computational time; however, annotated proteins of a majority of very large genomes were already available for search in the RefSeq database. Open reading frames >100 codons were extracted from the genome assemblies prior to BLAST searches. Predicted proteins from the Marine Microbial Eukaryote Transcriptome Sequencing Project (MMETSP) transcriptomes were downloaded from the iMicrobe server [20]. The documentation on the experimental conditions used during transcriptome acquisitions was obtained at the following internet adresses: [21] (MMETSP) and [22] (1KP).

Significant similarity and phylogenetic intertwining exist between packaging ATPase of NCLDVs and polintons, a family of large self-synthesizing transposons encoding up to 10 open reading frames [23]. To sort packaging ATPases between NCLDVs and polintons, a phylogenetic tree was constructed with all identified ATPases, NCLDV ATPases and polinton ATPases (reference polinton sequences were retrieved from the relevant supplementary data file of the Yutin et al. paper [23]). Identified ATPases that grouped with reference polinton homologs were removed from further study.

General phylogenetic analyses were performed as follows: additional homologous sequences were first searched in the RefSeq database using the BLAST EXPLORER tool [24]. Multiple-sequence alignment of homologous proteins was then performed using the MAFFT program [25]. We removed alignment positions containing >90% gaps before maximum likelihood phylogenetic reconstruction, which was performed using the FastTree program [26] with the LG + Gamma model of amino acid substitution. Statistical support for branches was estimated with the SH-like local support method. Sequences, alignments and phylogenetic trees are available in Dataset S1. The lengths of multiple-alignments used for phylogenetic reconstruction were 411, 1,198, 2,117, 692, and 565 amino acid positions (including position containing <90% gaps) for the ATPase, D5 primase-helicase, B-family DNA polymerase (DNAP), major capsid protein (MCP), and Very Late Transcription Factor 3 (VLTF3), respectively.

## 3. Results and Discussion

### 3.1. NCLDV Protein Markers in Eukaryotes

Although they typically encode hundreds of proteins, NCLDVs were reported to only share five universally-conserved core genes, including genes for MCP, D5 primase-helicase, DNAP, A32-like packaging ATPase, andVLTF3 [17]. These core viral proteins were used as query in BLAST searches against four eukaryotic sequence databases. The databases queried in this study included Genbank Refseq, which contained all annotated proteins from 680 sequenced eukaryotic species. In addition, we screened the Genbank Assembly database which contained 602 raw eukaryotic genome sequences that were not annotated and, therefore, not referenced in RefSeq. Open reading frames >100 codons were extracted from the non-annotated genome assemblies prior to their mining by BLASTP. Altogether, the RefSeq and Assembly databases comprised 1282 fully sequenced eukaryotic genomes. Because preliminary analysis revealed that NCLDV insertions were most frequent in aquatic unicellular eukaryotes, we also downloaded transcriptomic data from sequencing initiatives specifically targeting these organisms. The MMETSP database contained 679 assembled transcriptomes from 413 distinct marine unicellular eukaryotes, including some of the more abundant and ecologically significant species in the oceans such as diatoms [27]. The “1000 plants” (1KP) initiative database contained transcriptomic data from over 1000 plant species, including 214 unicellular eukaryotic algae from the Archaeplastida and Chromista groups [28].

Protein homologous to the five NCLDV core proteins were identified in 48 eukaryotic genomic sequencing projects, including 12 annotated genomes from RefSeq and 36 non-annotated genomes (Table 1). Nine of these genome assemblies contained the five core genes, while 14 genome assemblies contained only one of them. Some of the viral sequences arose from larger viral inserts that have already been described in the genomes of *Ectocarpus siliculosus* [12], *Bigelowiella natans* [8], *Physcomitrella patens* [9], *Acanthamoeba* spp. [13,16], *Hydra vulgaris* [10,13] and *Phytophthora parasitica* [10]. Most of the genomic datasets associated with NCLDV core protein homologs correspond to organisms living in soil or aquatic environments. The working genome dataset was highly dominated by Metazoa (*n* = 495), Fungi (*n* = 392) and land plants (*n* = 110), collectively representing 80% of the analyzed genomes. However, only 15% (7/48) of the eukaryotes positive for NCLDV proteins belonged to one of these groups, including three metazoans (*Daphnia pulex*, *H. vulgaris*, *Echinacea pallida*), three fungi (*Gonapodya prolifera*, *Rhizophagus irregularis*, *Allomyces macrogynus*), and a land plant (moss *P. patens*). Thus, a majority of eukaryotes associated with NCLDV proteins have a unicellular or simple multicellular structure and are members of less studied clades. The most impacted eukaryotic groups in terms of frequency are (i) brown algae (*Phaeophyceae*) for which all three genomes contained NCLDV homologs, Amoebozoa (11 out of 32 genomes = 34%), green algae (9/28 = 32%; i.e., *Chlorophyta* + *Streptophyta*), and *Oomycetes* (10/40 = 25%). As a matter of fact, small eukaryotes living either constantly (aquatic) or transiently (soil or swimming gametes (i.e., moss *P. patens*)) in waters appear to more frequently have integrated NCLDV sequences in their genome. This host bias may be a consequence of the relative large size of NCLDV particles, which makes their propagation more difficult out of fluidic environments. Also, the large virion size may limit propagation in complex multicellular organisms that have thick cell walls (i.e., terrestrial plants), giving them less chance to access the germ-line cells where lateral gene transfers must occur to be transmitted to the next host generation.

In addition, 18 transcriptomes generated from eukaryotic microalgae or aquatic protistans encode homologs to at least one of the NCLDV core proteins. In contrast to genomic datasets, none of the transcriptomes encode the five core proteins; 14 contained only one core protein sequence. DNAP was the most frequently identified NCLDV core gene among transcriptomes (10 species). This observation is consistent with a previous study reporting that a supernumerary DNAP subunit of possible NCLDV origin was transcribed in the rhizarian alga *B. natans*, whereas most of the other inserted NCLDV-like genes were transcriptionally silent [8]. The NCLDV-like DNAP of *B. natans* has been shown to be targeted to the nucleomorph where it might be involved in the nucleomorph genome replication [29]. Thus, some of the NCLDV-like DNAPs identified in these transcriptomes might also originate from lateral gene transfer from viruses and have acquired a functional role in their respective eukaryotes. Another possibility is that these transcripts were produced by viruses replicating in infected cells in the cultures used for sequencing. This is most likely the case for the *Emiliania huxleyii* viral transcripts because the corresponding transcriptomes have been reportedly acquired during a viral infection experiment (see information on the experimental conditions in Materials and Methods). In addition, the *Pleurochrysis carterae* strain sequenced in the MMETSP was suspected to contain a persistent virus, and our analysis gives credit to this hypothesis.

All in all, our study reveals many more potential NCLDV hosts than previously thought. Out of the 66 sequence datasets positive for NCLDV core genes, only four were generated from species already known for being infected by NCLDVs (i.e., *Acanthamoeba castellanii*, *Acanthamoeba polyphaga*, *E. siliculosus*, *E. huxleyii*). Two other species are closely related to organisms hosting NCLDVs. This is the case for the marine flagellate *Halocafeteria seosinensis* that is closely related to *Cafeteria roenbergensis* [30], a host for giant viruses and virophage [31,32]. The freshwater green alga *Chlorella vulgaris* is also closely related to *Craspedia variabilis* infected by *Paramecium bursaria* Chlorella viruses [33]. Overall NCLDV core proteins were identified in virtually all major groups of algae, including Chlorophyta, Streptophyta, Stramenopiles, *Cryptophyta*, Euglenozoa, Haptophyceae, and Rhizaria. Remarkably, NCLDV core proteins were identified from multiple species of a same genus such as *Acanthamoeba* spp., *Sphaeroforma* spp., *Phytophthora* spp., *Pythium* spp., *Klebsormidium* spp. and *Chlamydomonas* spp.

### 3.2. Phylogeny of Eukaryotic NCLDV-Like Proteins

**Figure 1:**
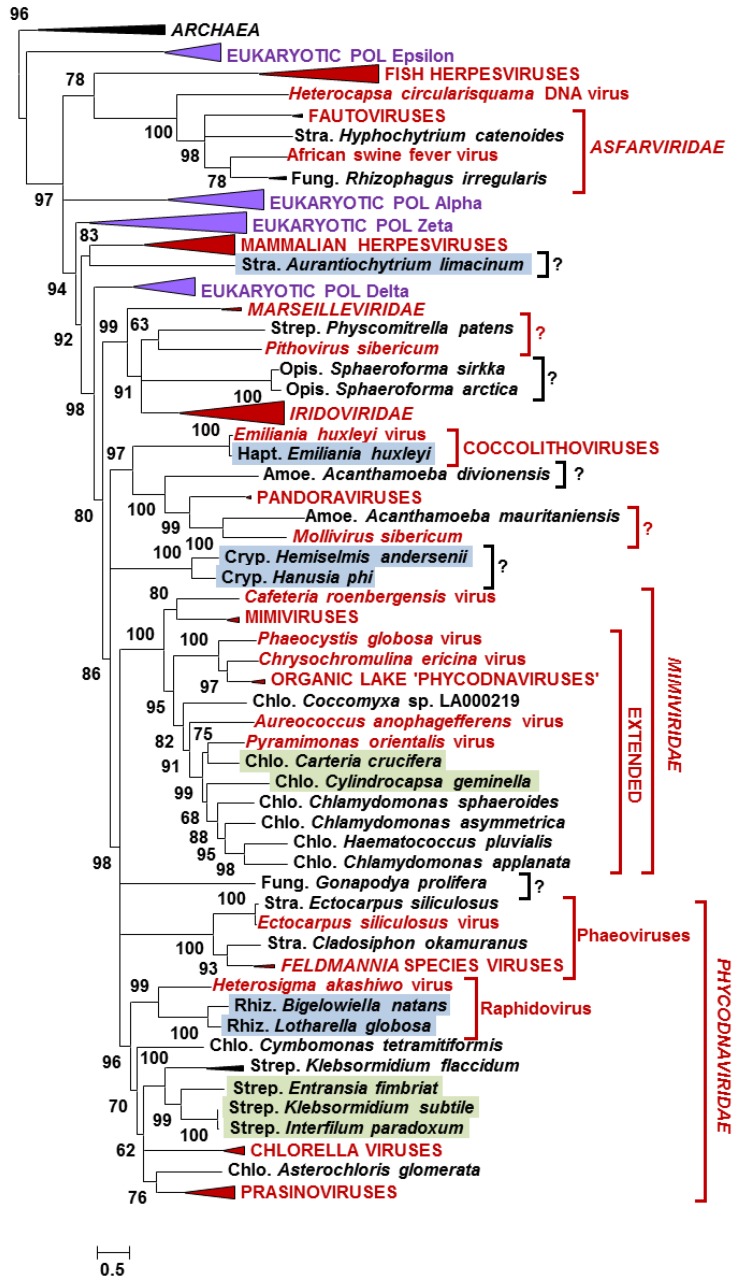
Maximum likelihood phylogenetic tree of DNA polymerase proteins. Statistical supports for branch (SH-like local support test) are given above or below nodes in percent. Branches with support less than 50% were collapsed. Species names with colored background indicate transcribed genes: green, 1KP transcriptomes; blue, MMETSP transcriptomes. Red and black question marks show potential extension of recognized viral groups or new viral clades, respectively. The scale bar indicates the number of substitution per site. Sequences, alignments, and phylogenetic trees are available in Dataset S1.

To investigate the phylogenetic relationships between NCLDV-like proteins identified in eukaryotic datasets and their homologs in extant viruses, maximum likelihood phylogenetic trees were constructed for each of the five NCLDV core proteins (Figure 1 and Figure S1–S4). Overall, the resulting phylogenetic trees revealed several general characteristics of the NCLDV-like sequences. First, most of the NCLDV core proteins identified in eukaryotic datasets branched close to or within existing viral clades, further supporting the hypothesis of their viral origin. Second, 20 eukaryotes listed in Table 1, containing two or more NCLDV core genes, occupied consistent positions across phylogenetic trees (i.e., grouped within the same viral clade in each phylogenetic tree). This observation suggests that the viral sequences in each eukaryote arose from a single unique virus rather than multiple unrelated viral sources. Lastly, closely related eukaryotes tended to share closely-related viral sequences. This involved organisms beyond the genus rank such as for example chlorophytan or streptophytan species which had sequences forming subtrees within the *Phycodnaviridae* or *Mimiviridae* clades, or Stramenopile species branching within the *Asfarviridae* clade. This phylogenetic “correlation” between virus sequences and potential hosts can occur if closely related virus-like sequences originate from a single viral genome integration event in an ancestral eukaryotic host—the transferred genes could then spread across the host progeny up to the extant species. It has been suggested that most *Acanthamoeba* inserted viral genes became nonfunctional and decayed by accumulation of mutations [16]; it is, therefore, possible that sequence homology can no longer be recognized between viral inserts after a sufficiently long period of divergence. Alternatively, some of these sequences may originate from closely related contaminating viruses. In fact, some viral clades are apparently specific to certain eukaryotic groups, such as for example chloroviruses that infect *Chlorella* species [33], prasinoviruses that infect prasinophyte algae [34], or phaeoviruses that infect brown algae [35], possibly because their speciation and diversification co-occurred with those of their hosts. Under this co-speciation scenario, closely related eukaryotes may be infected by closely related viral species, which can also result in the observations made in our phylogenetic trees.

The B-family DNAP gene is a core NCLDV gene traditionally used as a reference phylogenetic marker to establish the taxonomy of large DNA viruses [36,37]. The DNAPs of NCLDVs are phylogenetically related to those of eukaryotes [38]. Our phylogenetic tree is largely in agreement with previous studies and shows that the eukaryotic DNAP delta emerged as a sister group to most NCLDV DNAPs (Figure 1). Furthermore, *Asfarviridae*, together with fish herpesviruses and *Heterocapsa circularisquama* DNA virus, form a separate group from the other NCLDV DNAPs [38,39]. As expected, some of the virus-like DNAPs identified in algae branched within the *Phycodnaviridae* family, which is a group of NCLDVs exclusively infecting phytoplanktonic species [35]. Among these sequences were proteins from streptophytan and rhizarian species, two major algal clades for which no NCLDV has ever been reported so far. Moreover, virus-like DNAPs from seven chlorophytan species grouped within the “extended *Mimiviridae*” clade, another group of large DNA viruses infecting a variety of microalgae but more closely related to giant mimiviruses [40]. Interestingly these chlorophytes include three species of *Chlamydomonas.* This genus of green algae also contains *Chlamydomonas reinhardtii,* a model organism for molecular and chloroplast biology. Although 25 putative viral genes were identified in this species [11], none of the five NCLDV core protein genes were found in the *C. reinhardtii* genome. Finally, the non-photosynthtic stramenopile *Hyphochytrium catenoides* and the fungus *Rhizophagus irregularis* contained DNAP sequences branching within the *Asfarviridae*. This result suggests that viruses from the *Asfarviridae* family have a much wider host range than currently thought. Fungi have never been reported as a potential host for NCLDVs and the presence of a viral DNAP in *R. irregularis* and in another fungus *Gonapodya prolifera* (as well as a D5 primase-helicase in the fungus *Allomyces macrogynus;* Figure S3 and Table 1) suggest that these organisms may have been infected by members of NCLDVs. Note that *G. prolifera* and *A. macrogynus* are members of two ancestral fungal lineages, which contain species feeding on algae. Furthermore, the fungal D5 primase-helicases branched close to the *Heterosigma akashiwo* virus, which is a member of the *Phycodnaviridae*. Thus we cannot rule out that the NCLDV-like sequences identified in these two lower fungi may in fact originate from viruses infecting their algal preys.

Interestingly a number of virus-like DNAPs branched outside recognized taxonomic clades [41] suggesting that they belong to yet unknown taxa (e.g., indicated by black question marks in Figure 1). Other sequences grouping close to single, unclassified viral isolates (e.g., indicated by red question marks in Figure 1), might originate from members of the extended putative *Pithoviridae* family (e.g., represented by a sequence from the moss *P. patens*) and *Molliviridae* family (e.g., represented by a sequence from the amoeba *A. mauritaniensis*). Thus, our data, and more generally the approach of searching viral sequences in eukaryotic sequence data, make it possible to consolidate and even improve our knowledge of the NCLDV biodiversity. It is likely that some of the donor viruses encoded original functions and have developed new ways of interacting with their host that are radically different from the mechanisms already characterized.

### 3.3. Genomic Context around NCLDV-Like Genes

The discovery of viral sequences in eukaryotic genomes naturally poses the question of their origin, which can be a viral contamination, a provirus or a lateral gene transfer. A viral infection was suspected to be at the origin of the viral transcripts identified in the *E. huxleyii* and *P. carterae* transcriptomes. Filée suggested to examine the genomic environment around the virus-like genes to decipher whether they come from inserted viral DNA or contamination with free viral DNA during sequencing [13]. According to the author, insertion is the most likely hypothesis when virus-like genes are surrounded by intron-rich genes highly similar to eukaryotic homologs. We investigated the nature of genes surrounding NCLDV-like sequences in genomes that have a publicly available annotation, to the exception of organisms for which viral inserts have already been studied in details (i.e., *E. siliculosus* [12], *B. natans* [8], *P. patens* [9], *Acanthamoeba* spp. [13,16], *H. vulgaris* [10,13] and *P. parasitica* [10]). Figure S5 show the gene organization in contigs containing NCLDV core genes in seven eukaryotes, namely *A. macrogynus*, *D. pulex* [42], *E. pallida* [43], *Sphaeroforma arctica*, *Klebsormidium flaccidum* [44], *Saccharina japonica* [45], and *G. prolifera* [46].

Contigs with homologs to NCLDV core genes had sizes ranging from 1.2 kbp to 1.4 Mbp, so most of them contained more than one gene. The origin of the neighboring genes, inferred from the taxonomic information of their best match, generally indicates that other viral genes are present in the immediate vicinity of NCLDV core genes (Figure S5). Thus, most of the NCLDV core genes do not seem to result from horizontal transfer of a single isolated gene. However, this is not the case for the two metazoans, *D. pulex* and *E. pallida*, that each carries copies of a single NCLDV core gene (respectively, MCP and VLTF3) isolated in the midst of typical metazoan genes (Figure S5). Furthermore, the viral genes of the fungi *G. prolifera and A. macrogynus,* the brown alga *S. japonica* and the protist *S. arctica* are grouped in small genomic islands amid genes of eukaryotic origin, suggesting that they result from an insertion of a larger viral genome fragment. The viral sequences of *S. japonica* are closely related to phaeoviruses, which have a lysogenic reproduction and exist as provirus elements incorporated into the genomes of the brown algae *Ectocarpus siliculosus* and *Feldmannia* species [12,14,15]. Thus, the viral genes identified in the *S. japonica* genome might be remnants of an ancient provirus.

In contrast, NCLDV core genes of *K. flaccidum* are contained in contigs that have a dominance of viral genes, intermingled with a minority of genes most closely related to bacterial or eukaryotic homologs. Such a gene mosaicism is typical of NCLDV genomes [47]. Except for contig DF237168, there is no apparent juncture between a eukaryotic genomic region and a viral genomic region (i.e., a region containing a majority of eukaryotic genes followed by a region containing a majority of viral genes). Although this observation is compatible with a contaminating virus, this hypothesis is unlikely because a single viral genotype would be expected in the case of an infected culture. In contrast, we found five contigs containing a DNAP gene while NCLDVs only contain a single DNAP gene per genome. We also found six packaging ATPase genes, six MCP genes, and five VLTF3 genes. The levels of protein similarity between the DNAPs ranged from 40% to 92%, which excluded the possibility that the homologous regions originate from variants of a same initial viral genotype. Furthermore the gene order was highly rearranged between homologous regions, further refuting the hypothesis of a single viral genotype. These data suggest the *K. flaccidum* genome contains distinct viral insertions. These inserts may result from duplication of an original viral insert followed by sequence divergence and rearrangements of the duplicated copies. Alternatively, they may result from independent acquisitions from multiple viruses.

Altogether, our analysis of seven annotated eukaryotic genomes supports the hypothesis of lateral gene transfer from viruses rather than contamination with free viral DNA during sequencing. The same conclusion was drawn in other studies of various organisms including *Acanthamoeba* spp., *P. patens* and *B. natans* [8,9,13,16]. Thus, there is a general consensus indicating that when viral sequences are identified in a given eukaryotic genome assembly, they are likely to result from bona fide viral genomic insertions rather than an alternative source. Given the substantial number of eukaryotic genomes concerned by inserted viral sequences (Table 1), viruses, and especially NCLDVs, may soon take center stage in our understanding of eukaryotic genome evolution. Viral insertions may turn out to be a major force driving lateral gene transfers between viruses and eukaryotes or between eukaryotes. The wide phylogenetic spectrum of eukaryotes containing viral sequences also suggest that these inserts might serve as DNA template in an evolutionarily conserved defense mechanism against viruses based on sequence recognition such as the RNA interference (RNAi) pathway [48].

### 3.4. Hints on Viral Functions

Another interesting aspect of viral inserts is that genes contained within viral regions can provide hints on unexpected functional capabilities of the original viruses. For instance, we found two highly similar expansin genes in two NCLDV-like contigs of the *K. flaccidum* genome assembly (DF237168.1 and DF237869.1; Figure S5). Although most similar to plant homologs, these expansin genes are both surrounded by a VLTF3 gene and a hypothetical protein gene that only has homologs in phycodnaviruses. This suggests that the expansin genes had been captured by the original donor virus from a plant or algal cell, before lending to the *K. flaccidum* genome through integration of viral DNA. Expansins mediate cell wall extension in plants by disrupting non-covalent binding of wall polysaccharides [49,50]. Lateral transfers of expansin genes from plants toward their fungal and bacterial parasites have been described, and their functional similarity suggests that these proteins mediate plant-microbial interaction [51]. Thus, in analogy to cellular plant parasites, this gene could also have a role during viral infection by enabling the virus to cross the host cell wall barrier. This would consist in a case of functional convergence between eukaryotic, bacterial and viral plant pathogens.

Additionally, two *K. flaccidum* viral regions contained a gene encoding a U-type cyclin domain (DF237607.1 and DF237785.1; Figure S5). In the available *K. flaccidum* genome annotation the cyclin domain is predicted to be fused with a MCP domain. However, this protein structure is likely an annotation error resulting from the merging of two independent exons each containing one of the two domains. In fact, no transcript sequence supports the junction between the two introns in a *K. flaccidum* RNAseq study [44]. On the other side of the cyclin gene, we found a viral DNA packaging ATPase gene. Although the cyclin domain is more closely related to plant homologs, the viral origin of the surrounding genes suggests a cyclin gene captured from a plant cell was present in the original donor viral genome. Viral encoded cyclins have been identified in several viral families including herpesviruses, retroviruses, and baculoviruses, where they drive cell cycle transitions of the host [52]. Many DNA viruses induce quiescent cells to enter the cell cycle; this is thought to increase pools of deoxynucleotides and, thus, facilitate viral replication. In contrast, some viruses can arrest cells in a particular phase of the cell cycle that is favorable for replication of the specific virus [53]. If the existence of the predicted *K. flaccidum* virus is confirmed in future studies, it would represent the first instance of a cyclin gene in a NCLDV.

A contig of the fungus *G. prolifera* (KQ965906.1; Figure S5) contained a chitinase gene, together with three other viral genes encoding a MCP, a VATPase_H domain containing protein and a protein of unknown function. Chitinases are enzymes that degrade chitin, which is one of the most abundant biopolymers in nature. Chitin occurs in various contexts across a broad range of species and is the main constituent of fungal cell wall [54]. Remarkably, the *G. prolifera* chitinase is most closely related to homologs in chloroviruses (phycodnavirus) which are presumably involved in degradation of the cell wall of green algae of the *Chlorella* genus [55]. Interestingly the same *G. prolifera* viral region contains another chitinase-like protein surrounded by 2 viral genes, but this one is more similar to bacterial homologs. Thus, the putative *G. prolifera* virus might use a similar enzyme apparatus as chloroviruses to pass through the chitin-rich fungal cell wall.

## 4. Conclusions

We are only beginning to appreciate the extraordinary diversity of NCLDVs, which are among the most intriguing viruses on the planet. Here we show that substantial progress in the description of the NCLDV biodiversity can be made by mining potential host sequences in order to identify genetic markers of NCLDVs. Using this approach, both a virus and its putative host can be brought to light, a significant advantage over metagenomics, which cannot directly identify the two partners. This approach takes advantage of what appears to be an important, but as yet poorly understood, feature of NCLDVs: they leave footprints of their passage in the cell in the form of viral inserts in the host genome. Here we chose to search virus-like sequences using the five most consistently conserved genes in NCLDVs. Others suggested to use the RNA polymerase subunit 2 to identify giant viruses sequences in (meta)genomic data [10]. However, some of these genes are not universally conserved among NCLDVs. For instance, a RNA polymerase gene is absent in most *Phycodnaviridae* genomes, whereas a MCP gene could not be detected in the genomes of pandoraviruses and *P. sibericum* [3,56,57]. *P. sibericum* is also apparently lacking a gene for packaging ATPase. Thus additional combinations of NCLDV reference markers may lead to an increasing number of eukaryotic datasets positive for NCLDV sequences [11]. It is also worth noting that the abundance of viruses in environmental samples is sometimes estimated by quantitative PCR using primers specific for virus genes [58] or by the number of metagenomic reads overlapping viral genes [6]. However, given the apparent ease with which NCLDV genes find themselves integrated into host genomes, these approaches may lead to over estimating the viral abundances if the surveyed samples also contain hosts harboring viral HGTs.

In this study we could predict the existence of new members of NCLDVs, some of which are apparently distantly related from already characterized viruses and may define new viral clades. Examination of the gene content of viral regions also helped us predicting some potential functional capabilities of the original viruses. We also predicted a wide range of potential hosts, most of whom have never had an association described with NCLDVs. The validity of all these predictions must now be evaluated through experimental approaches. Most of the organisms in which viral sequences were found are cultivable in laboratory conditions. This offers a favorable experimental framework to prospect environmental samples in order to isolate viral strains by co-culture with a eukaryote. A potential host may be chosen according to the phylogenetic reconstruction of its viral sequences in order to target the isolation of novel NCLDVs of special scientific interest. If such an approach proves to be successful, it may help in improving our understanding of the NCLDV world.

## Figures and Tables

**Table 1:** Nucleocytoplasmic large DNA viruses (NCLDV) core protein homologs in eukaryotic sequence datasets.

Eukaryotic Clade	Species	Habitat	Database	DNAP *	MCP *	ATPase *	D5 *	VLTF3 *
**Genomic datasets**								
Amoebozoa (Discosea)	*Acanthamoeba astronyxis*	terrestrial and aquatic	Assembly	√		√		√
	*Acanthamoeba castellanii*		RefSeq		√	√	√	√
	*Acanthamoeba divionensis*		Assembly	√		√		√
	*Acanthamoeba healyi*		Assembly		√			
	*Acanthamoeba lenticulata*		Assembly		√	√	√	√
	*Acanthamoeba lugdunensis*		Assembly		√	√		
	*Acanthamoeba mauritaniens*		Assembly	√	√	√		√
	*Acanthamoeba pearcei*		Assembly			√	√	√
	*Acanthamoeba polyphaga*		Assembly			√	√	√
	*Acanthamoeba quina*		Assembly		√	√	√	√
	*Acanthamoeba rhysodes*		Assembly		√			
Cryptophyta (Pyrenomonadales)	*Guillardia theta*	sea	RefSeq					MI
Euglenozoa	*Euglena gracilis*	freshwater	Assembly		√			
Fungi (Chytridiomycota)	*Gonapodya prolifera*	freshwater	RefSeq	Phy	√	√	Phy	√
Fungi (Glomeromycota)	*Rhizophagus irregularis*	terrestrial	Assembly	Asf				
Fungi (Blastocladiomycota)	*Allomyces macrogynus*	freshwater	RefSeq				Phy	
Metazoa (Arthropoda)	*Daphnia pulex*	freshwater	RefSeq		√			
Metazoa (Cnidaria)	*Exaiptasia pallida*	sea	RefSeq					Asf
	*Hydra vulgaris*	freshwater	RefSeq		Mi	√	Mi	√
Opisthokonta (Ichthyosporea)	*Sphaeroforma arctica*	sea	RefSeq	Irma		Irma		
	*Sphaeroforma sirkka*	sea	Assembly	Irma	Irma	Irma	√	Irma
Rhizaria (Cercozoa)	*Bigelowiella natans*	sea	RefSeq	Phy + √	√	√	Phy	√
Stramenopiles (Bicosoecida)	*Halocafeteria seosinensis*	saltern pond	Assembly					√
Stramenopiles (Eustigmatophyceae)	*Nannochloropsis limnetica*	freshwater	Assembly		Pha			Pha
Stramenopiles (Hyphochytriomycetes)	*Hyphochytrium catenoides*	terrestrial	Assembly	Asf	Asf	Asf	Asf	Asf
Stramenopiles (Oomycetes)	*Phytophthora* sp*. totara*	soilborne plant pathogen	Assembly		Asf			
	*Phytophthora agathidicida*		Assembly		Asf			
	*Phytophthora alni*		Assembly					Asf
	*Phytophthora cambivora*		Assembly					Asf
	*Phytophthora cryptogea*		Assembly		Asf			
	*Phytophthora nicotianae*		Assembly		Asf			Asf
	*Phytophthora parasitica*		RefSeq		Asf			Asf
	*Pythium irregulare*		Assembly					Asf
	*Pythium oligadrum*		Assembly		Asf			
	*Pythium ultimum*		Assembly		Asf			Asf
Stramenopiles (Phaeophyceae)	*Cladosiphon okamuranus*	sea	Assembly	Pha	Pha	Pha	Pha	Pha
	*Ectocarpus siliculosus*		RefSeq	Pha	Pha	Pha	Pha	Pha
	*Saccharina japonica*		RefSeq		Pha			
Viridiplantae (Chlorophyta)	*Asterochloris glomerata*	lichen photobiont	Assembly	Phy	Phy	Phy	Phy	
	*Chlamydomonas applanata*	terrestrial	Assembly	Mi				
	*Chlamydomonas asymmetrica*	freshwater	Assembly	Mi	Mi	Mi	Mi + Phy	Mi
	*Chlamydomonas sphaeroides*	freshwater	Assembly	Mi	Mi	Mi	Mi + Phy	Mi + √
	*Chlorella vulgaris*	freshwater	Assembly		√			
	*Coccomyxa* sp*. LA000219*	unknown	Assembly	Mi	Mi	Mi	Mi	Mi
	*Cymbomonas tetramitiformis*	sea	Assembly	Phy	Phy	Phy	Phy	Phy
	*Haematococcus pluvialis*	freshwater	Assembly	Mi	Mi	Mi	Mi + Phy	Mi
Viridiplantae (Streptophyta)	*Klebsormidium flaccidum*	terrestrial	RefSeq	Phy	Phy	Phy	√	Phy
Viridiplantae (Streptophyta)	*Physcomitrella patens*	terrestrial	RefSeq	Pitho			Pitho	
**Transcriptomic datasets**								
Cryptophyta (Cryptomonadales)	*Hemiselmis andersenii*	sea	MMETSP	√				
Cryptophyta (Pyrenomonadales)	*Hanusia phi*	sea	MMETSP	√				
Haptophyceae (Coccolithales)	*Pleurochrysis carterae*	sea	MMETSP		√	√		√
Haptophyceae (Isochrysidales)	*Chrysochromulina polylepis*	sea	MMETSP			√		
	*Isochrysis galbana*	sea	MMETSP			√		
Haptophyceae (Phaeocystales)	*Phaeocystis antarctica*	sea	MMETSP		√			
Haptophyceae (Prymnesiales)	*Emiliania huxleyi*	sea	MMETSP	Coc	Coc		Coc	
Rhizaria (Cercozoa)	*Lotharella globosa*	sea	MMETSP	Phy				
Stramenopiles (Labyrinthulomycetes)	*Aurantiochytrium limacinum*	sea	MMETSP	Pha				
	*Schizochytrium aggregatum*	sea	MMETSP		√			√
	*Thraustochytrium* sp.	sea	MMETSP		√			√
Undescribed Strain	*CCMP2135*	sea	MMETSP					√
Undescribed Strain	*CCMP2436*	sea	MMETSP				√	
Viridiplantae (Chlorophyta)	*Carteria crucifera*	freshwater	1KP	Mi			Mi	
	*Cylindrocapsa geminella*	freshwater	1KP	Mi				
Viridiplantae (Streptophyta)	*Entransia fimbriat*	freshwater	1KP	Phy				
	*Interfilum paradoxum*	terrestrial	1KP	Phy				
	*Klebsormidium subtile*	terrestrial	1KP	Phy				

* putative phylogenetic grouping of the NCLDV core protein homologs based on the phylogenetic trees presented in Figure 1 and Figure S1–S4: √ = unknown clade, Phy = *Phycodnaviridae*, Mi = *Mimiviridae*, Pha = phaeoviruses, Coc = coccolothoviruses, Pitho = putative Pithoviridae, Asf = *Asfarviridae* and IrMa = *Iridoviridae*/*Marseilleviridae* cluster. Column names: DNAP, DNA polymerase; MCP, major capsid protein; ATPase, DNA packaging ATPase; D5, D5 helicase; VLTF3, very late transcription factor 3.

## Supplementary Materials

The following are available online at www.mdpi.com/1999-4915/9/1/17/s1, Figure S1: Maximum likelihood phylogenetic tree of MCPs, Figure S2: Maximum likelihood phylogenetic tree of packaging ATPases, Figure S3: Maximum likelihood phylogenetic tree of D5 helicase-primase, Figure S4: Maximum likelihood phylogenetic tree of VLTF3, Figure S5: Genomic context of the NCLDV core genes identified in eukaryotic annotated genomes, Dataset S1: Alignments and phylogenetic trees of the 5 NCLDV core proteins and their eukaryotic homologs.
