# Supplementary material for: A Glimpse of Nucleo-Cytoplasmic Large DNA Virus Biodiversity through the Eukaryotic Genomics Window
Source: Viruses. 2017 Jan 20;9(1):17. doi: 10.3390/v9010017 (PMC5294986; doi:10.3390/v9010017)
Supplement: Supplementary file 1 [file viruses-09-00017-s001.pdf]

Supplementary Materials: A Glimpse of Nucleo-Cytoplasmic Large DNA Virus Biodiversity through the Eukaryotic Genomics Window

Lucie Gallot-Lavallée [1] and Guillaume Blanc [1,2,*]

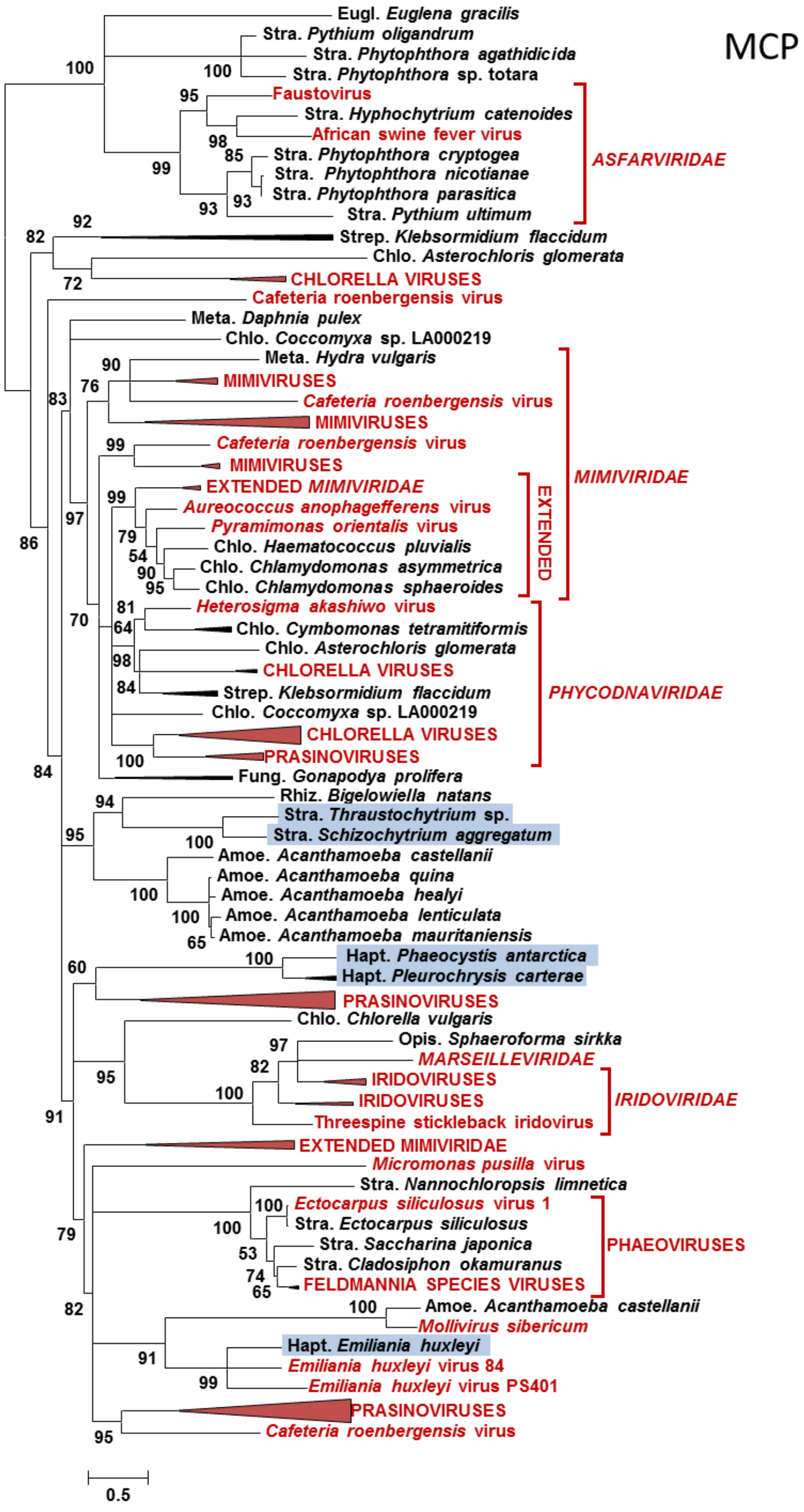

Figure S1. Maximum likelihood phylogenetic tree of MCPs. The figure legend is same as in Figure 1.

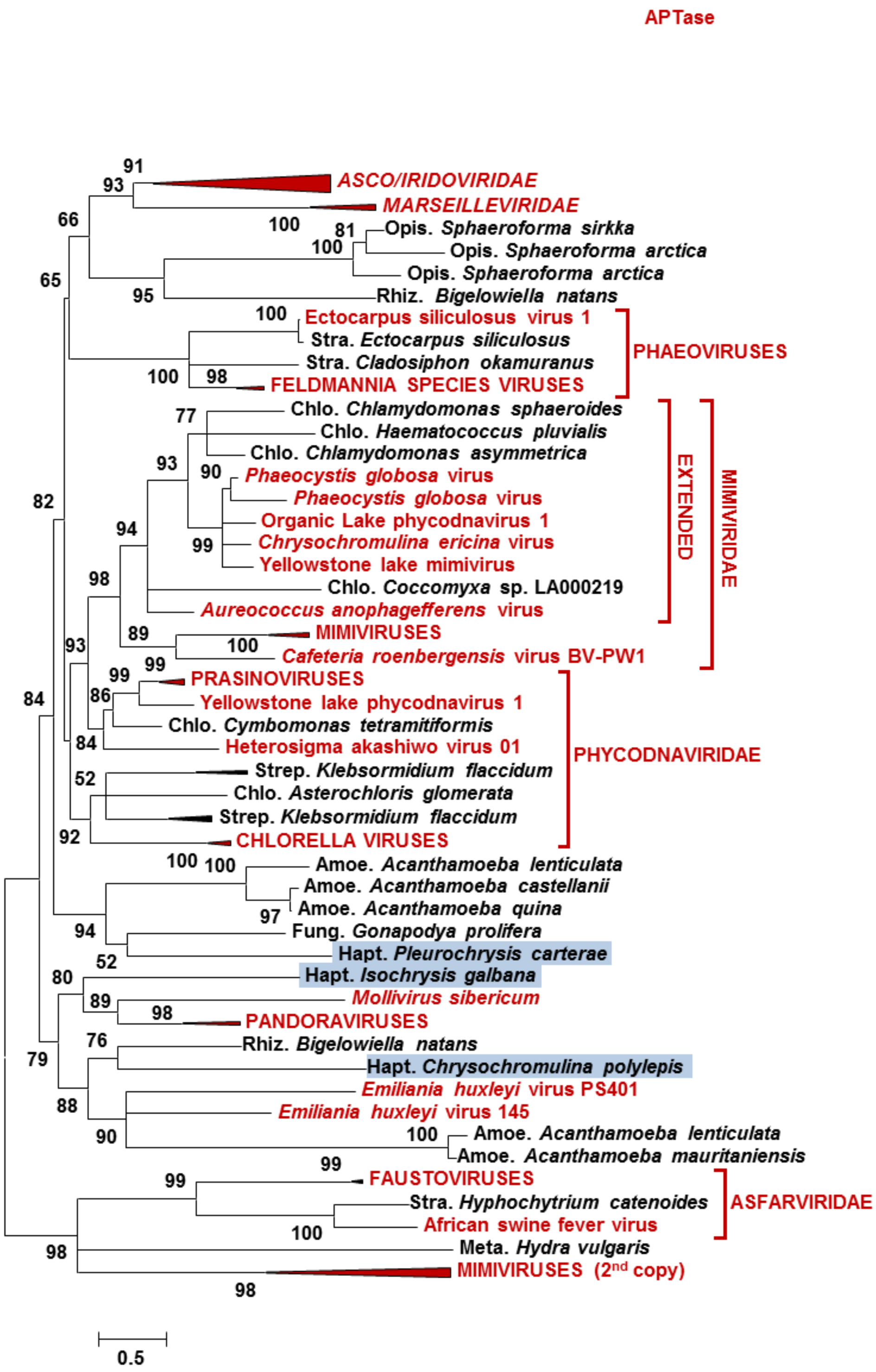

Figure S2. Maximum likelihood phylogenetic tree of packaging ATPases. The figure legend is same as in Figure 1.

D5

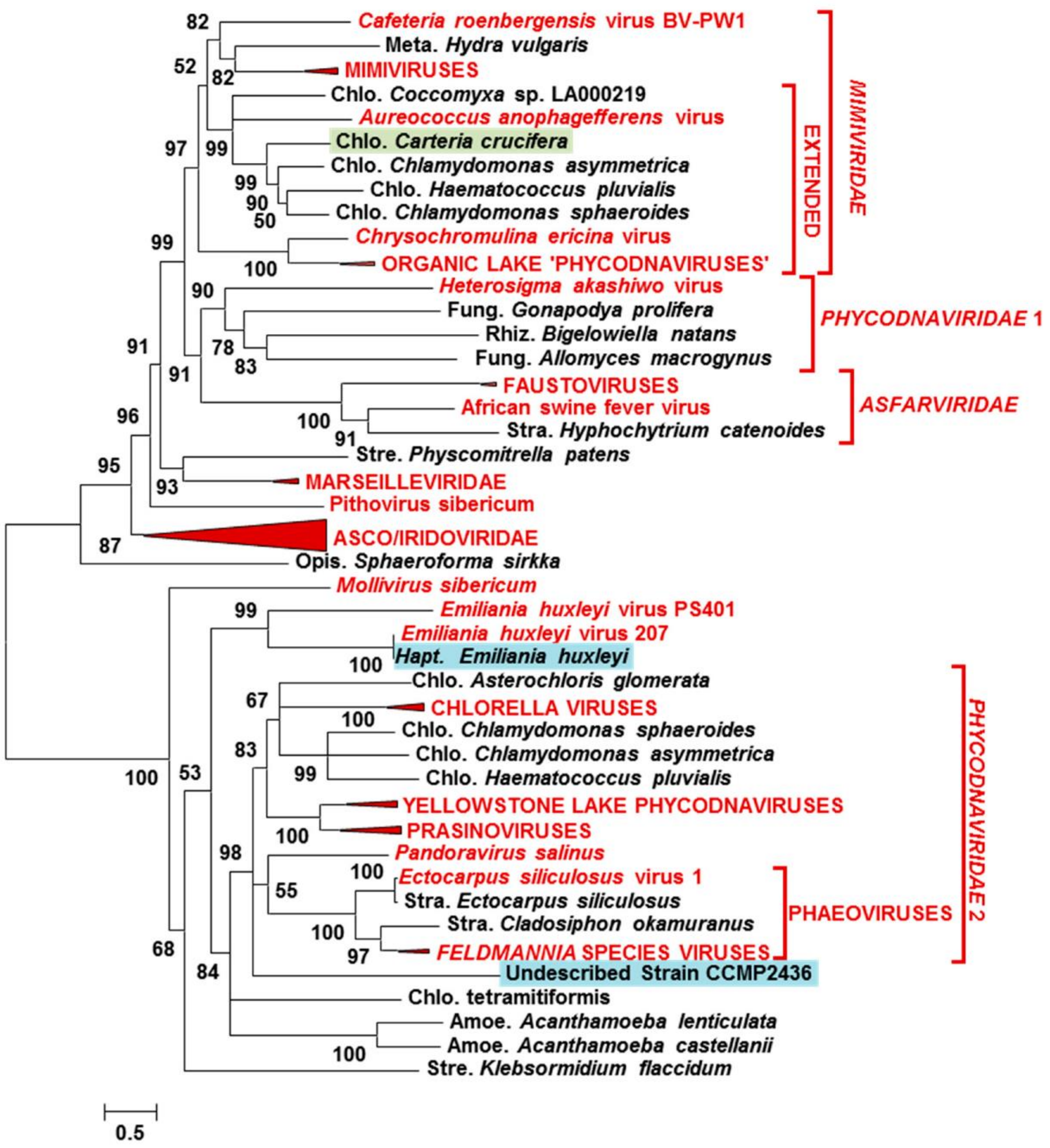

Figure S3. Maximum likelihood phylogenetic tree of D5 helicase-primase. The figure legend is same as in Figure 1.

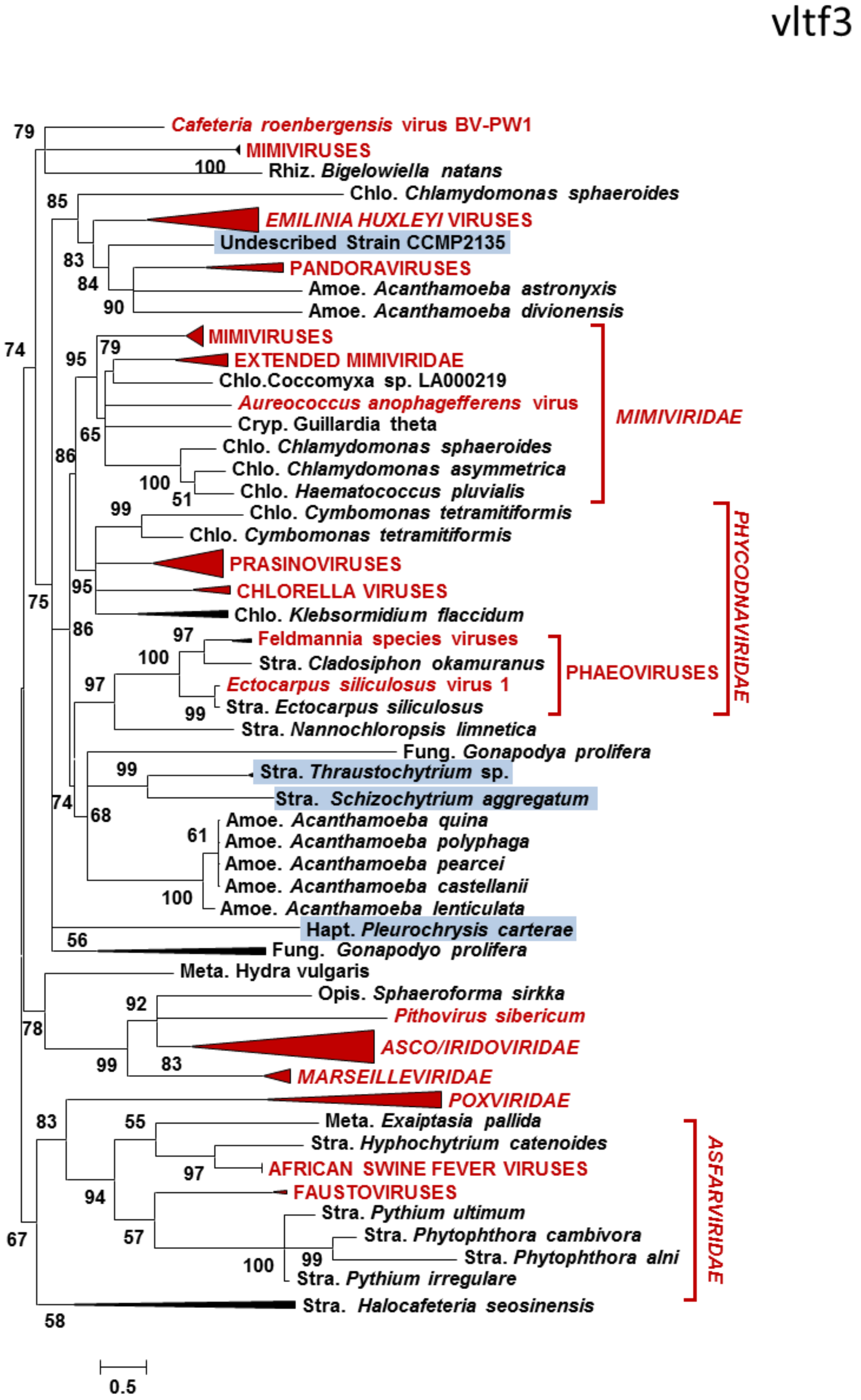

Figure S4. Maximum likelihood phylogenetic tree of VLTF3. The figure legend is same as in Figure 1.

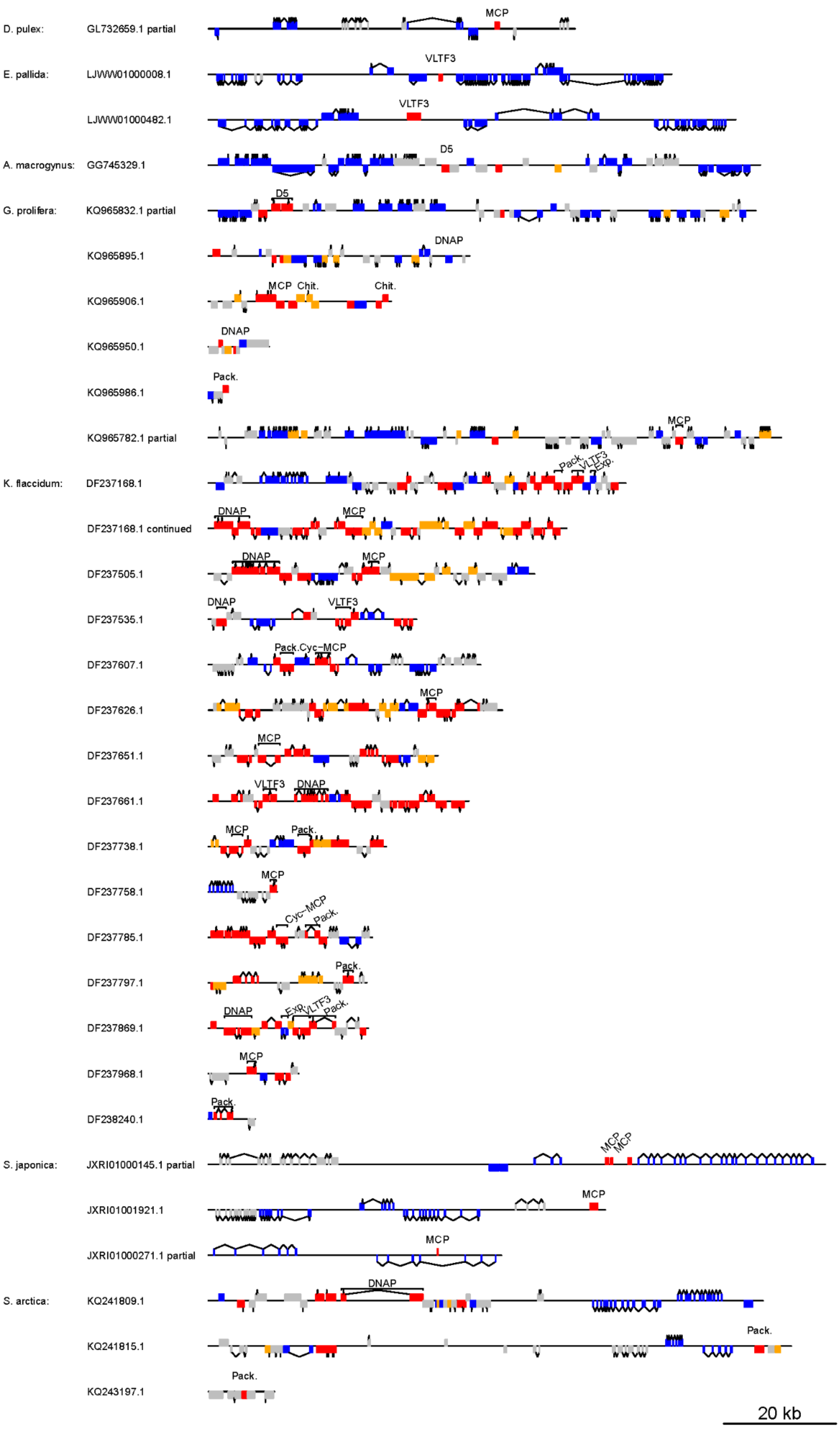

Figure S5. Genomic context of the NCLDV core genes identified in eukaryotic annotated genomes. Genbank gene annotations and viral genes detected in this study are represented by colored rectangles along the contigs. The colors indicate the taxonomic assignation of the gene's best match: blue, eukaryotes; orange, prokaryote; red, NCLDV; gray, no match. Interesting genes discussed in the manuscript are flagged as follows: MCP: major capsid protein; DNAP: DNA polymerase; Pack.: DNA packaging ATPase; D5: D5 helicase-primase; VLTF3: very late transcription factor 3; Cyc: cyclin; Exp.: expansin; Chit.: chitinase.

© 2017 by the authors. Submitted for possible open access publication under the terms and conditions of the Creative Commons Attribution (CC BY) license (http://creativecommons.org/licenses/by/4.0/).
